# A proposed simulation method for directed self-assembly of nanographene

**DOI:** 10.1088/1361-648X/aa7c0b

**Published:** 2017-08-01

**Authors:** J A Geraets, J P C Baldwin, R Twarock, Y Hancock

**Affiliations:** 1 Department of Physics, University of York, Heslington, York YO10 5DD, United Kingdom; 2 Department of Biology, University of York, Heslington, York YO10 5DD, United Kingdom; 3 York Centre for Complex Systems Analysis, University of York, Heslington, York YO10 5GE, United Kingdom; 4 Department of Mathematics, University of York, Heslington, York YO10 5DD, United Kingdom; y.hancock@york.ac.uk

**Keywords:** nanographene, kinetic self-assembly, chemical synthesis, nanodevice design, computational methods

## Abstract

A methodology for predictive kinetic self-assembly modeling of bottom-up chemical synthesis of nanographene is proposed. The method maintains physical transparency in using a novel array format to efficiently store molecule information and by using array operations to determine reaction possibilities. Within a minimal model approach, the parameter space for the bond activation energies (i.e. molecule functionalization) at fixed reaction temperature and initial molecule concentrations is explored. *Directed* self-assembly of nanographene from functionalized tetrabenzanthracene and benzene is studied with regions in the activation energy phase-space showing length-to-width ratio tunability. The degree of defects and reaction reproducibility in the simulations is also determined, with the rate of functionalized benzene addition providing additional control of the dimension and quality of the nanographene. Comparison of the reaction energetics to available density functional theory data suggests the synthesis may be experimentally tenable using aryl-halide cross-coupling and noble metal surface-assisted catalysis. With full access to the intermediate reaction network and with dynamic coupling to density functional theory-informed tight-binding simulation, the method is proposed as a computationally efficient means towards detailed simulation-driven design of new nanographene systems.

## 1. Introduction

Nanographene has immense potential for technological applications [1]. For example, graphene nanoribbons have band gaps dependent on ribbon-width, chemical functionalization, patterning and edge-structure [2, 3], with potential applications as logic transistors, quantum dot structures and in optoelectronics [4]. Although nanographene properties allow for the design of future miniaturized devices, progress towards their realization is limited by a lack of atomic-scale control in top-down fabrication. To assist with these limitations, bottom-up chemical synthesis experiments have been proposed for producing nanographene with atomically precise edges and patterning, e.g. Cai *et al* [5]. Such experiments involve a two-step, surface-assisted process of dehalogenation of precursor polyaromatic hydrocarbon molecules and cyclodehydrogenation, although other chemical synthesis routes have also been explored [6, 7].

Modeling the bottom-up chemical synthesis of nanographene allows for an understanding of underlying chemical and physical processes, which may then inform experiment. Previous work on carbon systems has focussed on the reaction energetics of chemical synthesis steps using density functional theory for specific surfaces and carbon-based molecules (e.g. Blankenburg *et al* [8]). In modeling self-assembly processes, molecular-dynamics [9] and kinetic Monte Carlo [10, 11] have also been used for specific precursor molecules, surfaces and reaction conditions. In this paper, a physically transparent method is proposed, which uses a model-system approach and exploits the selective functionalization of precursor polyaromatic hydrocarbon molecules for *directed* nanographene kinetic self-assembly. The advantage of the kinetic self-assembly approach is that it provides a theoretical framework to identify simple sets of rules underpinning molecular assembly that are not easily probed using kinetic Monte Carlo or molecular dynamics methods.

Predictive modeling requires flexibility to account for various precursor molecules and reaction pathways. Already several reaction types can lead to a variety of graphene nanostructures, including complex systems such as graphene nanoribbons with added atom decoration [12]. One benefit of a predictive kinetic self-assembly approach is that other, yet-to-be explored synthesis routes can be simulated. For example, the Ullmann reaction [13] that is used in Han *et al* [14] provides a route to both symmetric and asymmetric couplings. However, greater versatility in nanographene products can also be obtained using asymmetric Suzuki–Miyaura couplings, which allow for different reaction conditions and energetics (i.e. catalysts, etc) [13]. Thus, there exists several types of aryl–aryl couplings for designer *click-chemistry* synthesis. A further benefit of predictive simulation is the ability to explore a parameter space for the reaction conditions allowing for materials discovery via directed synthesis. For example, the parameter space can be investigated to suggest reactants and reaction conditions for self-assembly products with desired length-to-width ratios. Specific to the kinetic self-assembly method, intermediate products and reaction networks can also be interrogated to determine the most energetically favorable pathway for directed nanographene design.

A novel feature of the proposed self-assembly method is that it maintains physical transparency by using matrix arrays to store the precursor, intermediate and product molecular structures. These arrays exploit the base symmetries of the molecules and can be easily manipulated using matrix operations (rotations, translations, etc) to generate complex networks of reaction possibilities, which are then stored using reciprocal space compression. All of these features allow for a predictive model that is efficient and can be modified to include different precursors, surfaces and types of reactions.

**Figure 1.**
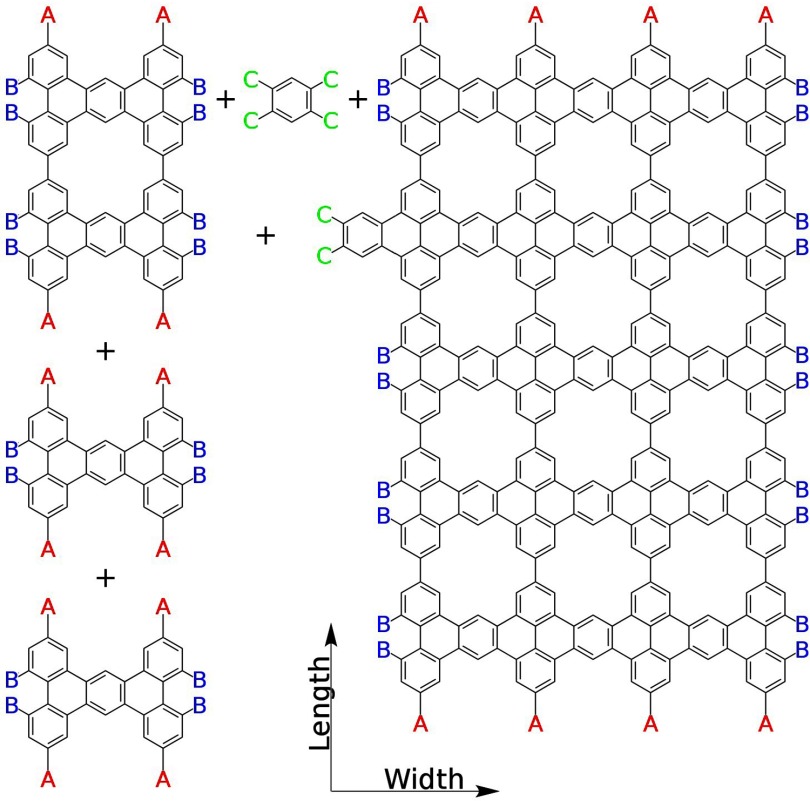
Example of a proposed *click-chemistry* synthesis used in this work for directed kinetic self-assembly modeling. Functionalized tetrabenzathracene and benzene precursor molecules couple to produce nanopatterned graphene via A–A (symmetric) and B–C (asymmetric) coupling reactions.

To demonstrate the predictive kinetic self-assembly method, a simple test case is explored of functionalized tetrabenzanthracene and functionalized benzene synthesis of nanographene with atomic-scale patterning (figure 1). Chemical point-functionalization of tetrabenzanthracene and benzene is well established, e.g. Artal *et al* [15] and Freeman *et al* [16], and the assembly of tetrabenzanthracene systems is also of interest [17]. For the purpose of detailing the computational method, the exemplar study assumes a minimal model approach. In this respect, ranges for the bond activation energies are explored, with surface catalysis effects included to first order. The reactions are also assumed to be coupling-limited with this deemed plausible against chemical synthesis and DFT studies that show the rate-limiting step to be de-functionalization of the precursor molecules [6]. Although the details of the surface are not explicitly considered, these can later be added as an extension. For example, the energetics for the kinetic self-assembly modelling can be expanded to included specific surface-molecule interactions determined via density functional theory simulations.

**Figure 2.**
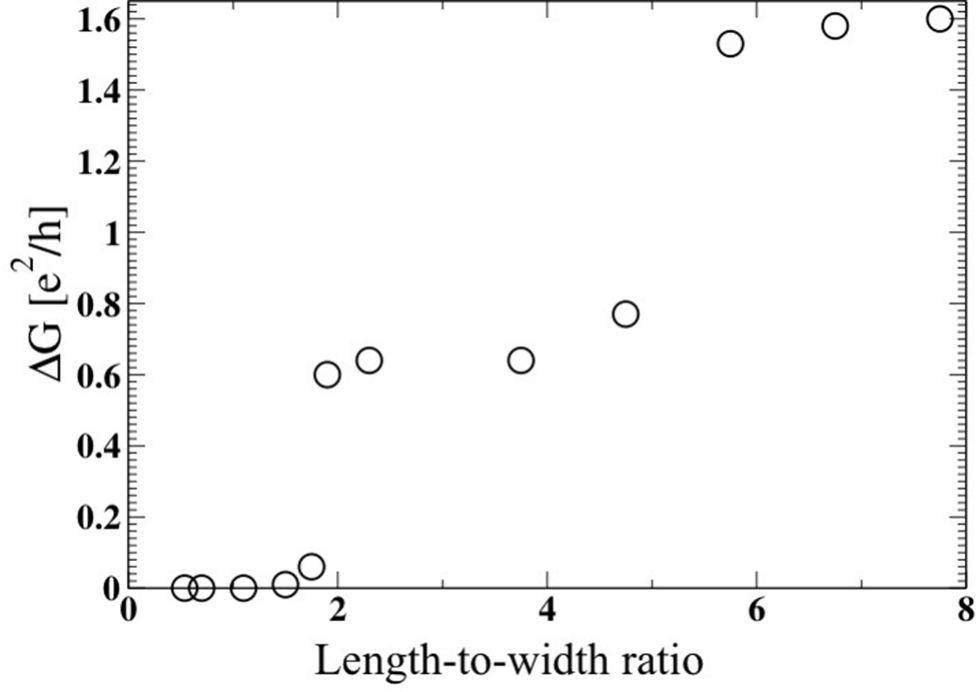
The conductance gap ($\Delta G$) in units of the quantum conductance ($e^2/h$) as a function of the device length-to-width ratio for the armchair-edge device (figure 1) with hydrogen edge-passivation. These coherent transport results were calculated using a generalized tight-binding model [18].

The specific choice of nanographene self-assembly (figure 1) is further motivated by coherent transport results for armchair-edge devices of this kind that have hydrogen edge-passivation. These results, obtained using a density functional theory-informed, generalized tight-binding method [18], show a tunable conductance gap as a function of the device length-to-width ratio (figure 2). The formation of band gaps and conduction gaps is expected in patterned systems due to the loss of conduction channels arising from the patterning, with these effects previously reported for patterned and vacancy-defected nanographene [20, 21]. The possibility to tune the conductance gap, as evidenced here, motivates the need to efficiently develop new synthesis methods for the controlled production of novel nanographene products that are of good quality and have specific features (such as the length-to-width ratio, atomic-scale patterning, etc). We will use the test case in this work (figure 1) to show that the proposed kinetic self-assembly approach is an efficient and transparent means of determining the possible chemical synthesis energetics, experimental conditions and reaction pathways to achieve these aims. Further, we will demonstrate how the model parameterization can be compared against available data to then propose a viable type of aryl-functionalization and catalyst to obtain optimised synthesis of nanographene with length-to-width ratio tunability.

## 2. Computational method

### 2.1. Matrix formalism

**Figure 3.**
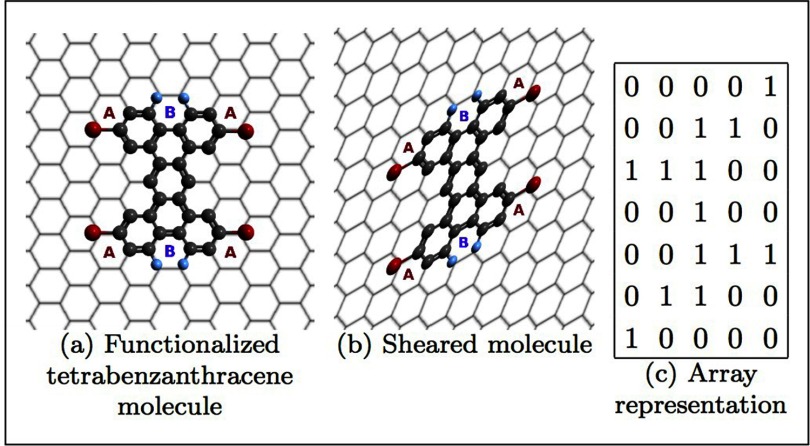
(a) A functionalized tetrabenzanthracene precursor molecule with A- and B-type point-functionalizations. The honeycomb lattice is sheared (b), and translated into the array representation (c). The number 1 in the array corresponds to one of the three constituent sub-molecule groups defined for this system, namely the functionalized sites A and the two adjacent B sites (the latter assumed to react simultaneously), as well as the benzene ring components.

Efficient and physically transparent kinetic self-assembly modeling is proposed by representing each molecule (precursors, intermediates and products) by an array. As an example of its use, figure 3 shows the array representation for a functionalized tetrabenzanthracene structure with its symmetry encapsulated in a sheared honeycomb lattice representation. In this *full structure* array, the molecules are succinctly described by sub-molecule groups pertaining to the A reaction sites, the two adjacent B reaction sites (considered together) and the benzene components, rather than by individual atomic positions. The sub-molecule groups are represented by the number 1 in the array, with the two adjacent B reaction sites assumed to react simultaneously.

**Figure 4.**
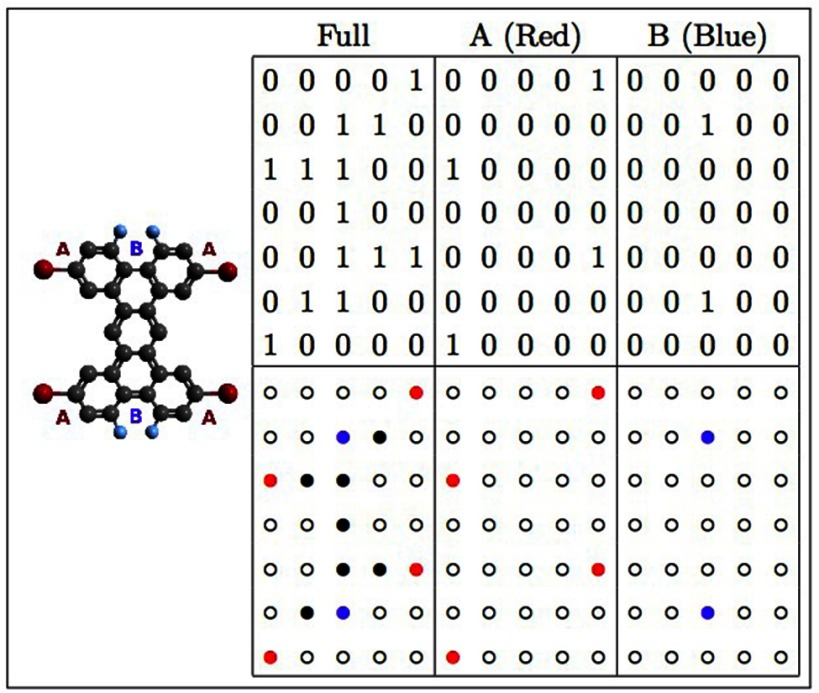
A functionalized tetrabenzanthracene molecule (left) with arrays representing the full structure (Full), reaction sites A (Red) and the two adjacent reaction sites B (Blue) (top right). The symbolic molecular structures are shown underneath.

**Figure 5.**
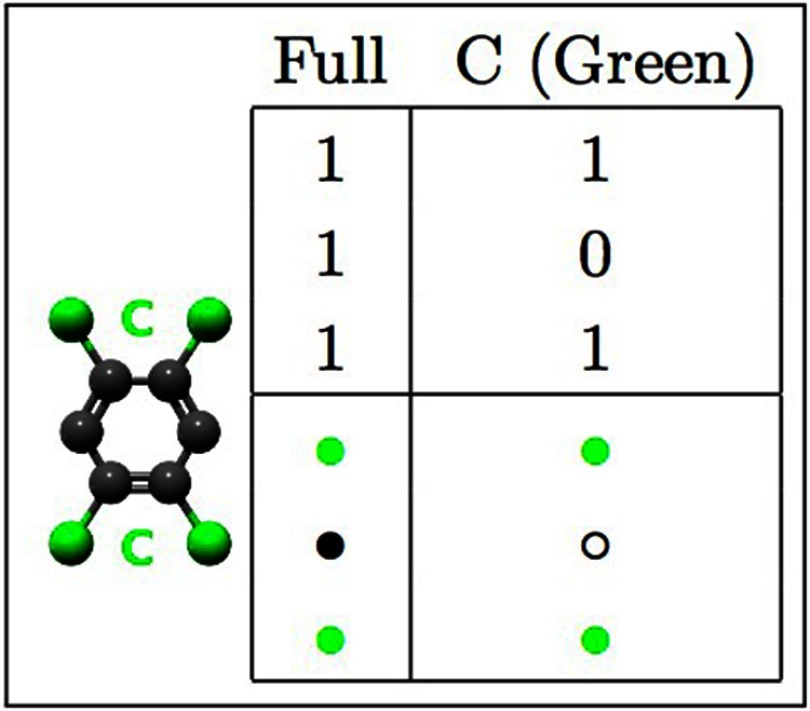
A functionalized benzene molecule (left) and arrays (top) representing the full structure (Full), and the reaction site array (C). The two adjacent reaction sites, C (assumed to react simulataneously), are represented as a single unit in the arrays. Symbolic representations of the sub-molecule components are shown underneath.

In addition to the full structures, arrays are also constructed for the molecular reaction sites so that these can be tracked. Here, figures 4 and 5 show the full structure and reaction site arrays for the functionalized tetrabenzanthracene and benzene structures, respectively. Symbolic representations of these arrays (also shown in figures 4 and 5) provide condensed and physically transparent storage of the essential molecule sub-groups (i.e. reaction sites and benzene components). The array representation allows for lattice transformations commensurate with the array coordinates, e.g. molecular rotations in increments of 30°, translations, as well as 2-fold plane inversion transformations. These operations, and the cross-correlation of arrays, determine possible molecule–molecule orientations, interactions and relative positions in which the molecules can form bonds.

**Figure 6.**
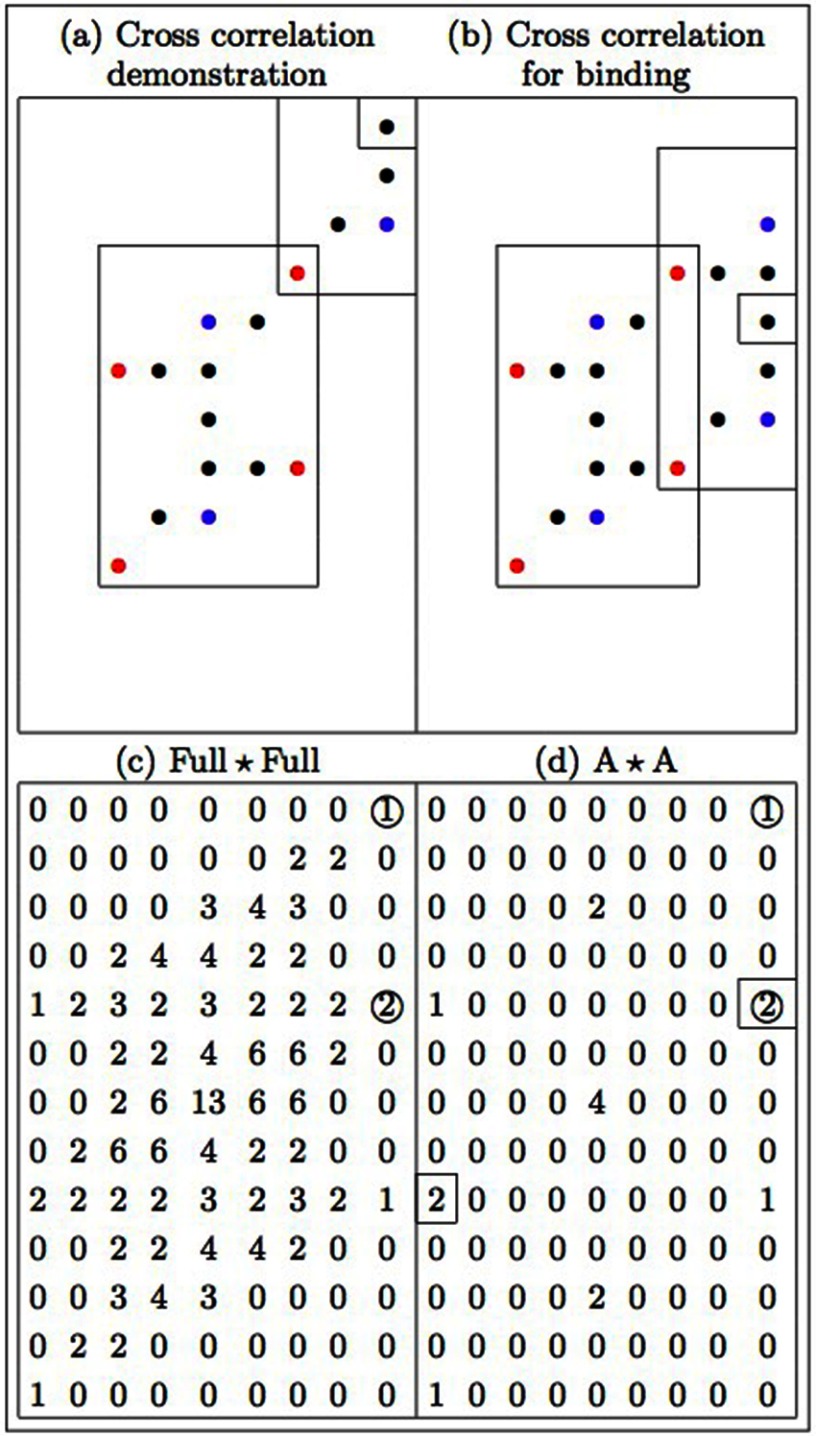
Autocorrelation between two tetrabenzanthracene arrays showing one molecule translated over another. There occurs one A–A overlap in (a) and two A–A overlaps in (b). The numbers 1 and 2 that are circled in the autocorrelation arrays (c) and (d) are representative of these events. An agreement between array elements in (c) and (d) represent allowed binding events, such as the A–A bindings associated with the values of 2 highlighted by a square in (d). *N.b*. single bond binding events such as in (a) are not permitted due to molecule rotation (steric hinderance).

The array functionality in the kinetic self-assembly model is illustrated in the bonding of two tetrabenzanthracene molecules via the autocorrelation of the full structure (${\rm Full \star Full}$) and reaction site (${\rm A \star A}$) tetrabenzanthracene arrays (figure 6). In this example, a sliding window translates one molecule over the other, from top to bottom and from left to right. Snapshots of two possible translations showing the overlap of the A–A reaction sites are given (figures 6(a) and (b)). For the single A–A overlap event in figure 6(a), a value of 1 is recorded in the autocorrelation arrays (circled) and, for the double A–A overlap event in figure 6(b), a value of 2 is recorded in the autocorrelation arrays (circled) (figures 6(c) and (d), respectively). The value of 13 in the centre of the Full$\star$Full array represents the event where the two molecules are fully superimposed. In figure 6(d), a corresponding value of 4 in the centre of the A$\star$A array denotes the number of overlaps of the A–A reaction sites associated with the full superposition of the two molecules.

The mathematical description of this array functionality involves the circular cross-correlation between two molecular structure arrays, which for arbitrary molecular structures *f* and *g* is

1 \begin{align*} \displaystyle ^{{\rm circ}}\{\,f\star g\}(i,j)=\sum^{M-1}_{m=0}\sum^{N-1}_{n=0}f(m,n)g(m-i,n-j). \nonumber \end{align*}

Here, *M* is the maximum width and *N* is the maximum height of the *f* and *g* matrices having array indices *i* and *j*, and $M\times N$ is the size of the output matrix. More succinctly, the circular cross-correlation can also be obtained using discrete Fourier transforms and by applying the correlation theorem [19], such that

2 \begin{align*} \displaystyle ^{{\rm circ}}\{\,f\star g\}(i,j)=\mathcal{F}_D^{-1}[F^{\ast}(\,p,q)G(\,p,q)] \nonumber \end{align*}

where $F(\,p, q)=\mathcal{F}_D^{-1}[\,f(i, j)]$ and $G(\,p, q)=\mathcal{F}_D^{-1}[g(i, j)]$. Here, $\mathcal{F}_D$ and $\mathcal{F}_D^{-1}$ are the discrete direct and indirect Fourier transforms, respectively, and *p* and *q* are indices in phase space. Using a reciprocal space representation provides a computationally efficient means of storing detailed molecular information, full reaction history, intermediates and the final reaction products.

Following the circular cross-correlation procedure, all possible binding events between the two molecules are determined before binding with a third party molecule can be considered. Binding between molecules where two bonds form is permitted as this event prevents molecular rotation, with single bonds disallowed to prevent steric hinderance. For the two tetrabenzanthracene molecules in this example (figure 6), possible A–A binding events have been identified by numerical matches between elements in the cross-correlated Full$\star$Full and the A$\star$A binding arrays (see figures 6(c) and (d)). Thus, only the double-bond binding events indicated by the number two (highlighted with a square), which match in the Full$\star$Full and A$\star$A arrays, are allowed.

One advantage of the array method is that intermediate arrays (i.e. product structures from binding events) can be stored and tracked throughout the simulation. The intermediate arrays are created from any permitted binding event, such as the one shown in figure 6(b), and are easily accessible. For example, assume that $f\star g(i, j)$ results in a binding event at $(a_i, a_j)$ due to a match between the full and binding arrays at this coordinate position. Translating the array element $g(i, j)$ by $(a_i-1, a_j-1)$ will recreate the overlap sliding window for that element. By then applying this translation to each element in the *g* molecule structure array,

3 \begin{align*} \displaystyle g(i,j) \rightarrow g(i+a_i-1,j+a_j-1), \nonumber \end{align*}

the co-joined molecules, and hence an intermediate product structure array, can then be produced from

4 \begin{align*} \displaystyle f(i,j)\cup g(i+a_i-1,j+a_j-1). \nonumber \end{align*}

This procedure can also be applied to the binding arrays.

**Figure 7.**
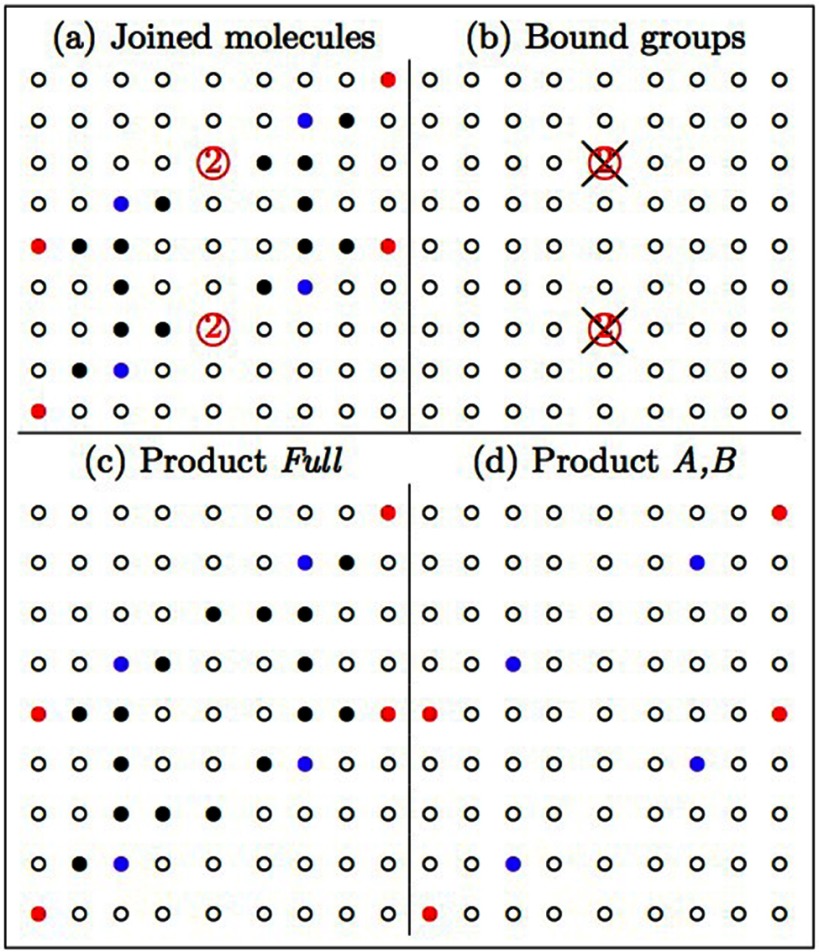
Intermediate molecule obtained from the binding event in figure 6(b). The joined precursor molecules are described by a larger combined array (a). The interacting functional groups for the binding event (b) are replaced by the number one (i.e. black dots) in the Full (product molecule) array (c) and removed from the product (A) and (B) binding array (d).

An example showing the intermediate structure generated from the A–A binding event between two tetrabenzanthracene molecules (figure 6(b)) is given in figure 7. To obtain the intermediate full molecule array from the symbolic structure (product full, figure 7(c)), all of the nonzero elements, including the bound sites (figures 7(a) and (b)) are reassigned to one. In the functional group array, all elements greater than one are removed as the binding positions have been used (figure 7(d)).

### 2.2. Kinetic self-assembly modeling

The reacting system evolves via the Arrhenius equation

5 \begin{align*} \displaystyle k = A_{o}{\rm e}^{-E_a / ({k_{\rm B} T})} \label{Arrhenius} \nonumber \end{align*}

where in the example system, $k=k_{AA}$ and $k_{{\rm B}C}$ are the rate constants for the A–A and B–C reactions, respectively (figure 1). Here, $A_{o}=10^9~$${\rm s}^{-1}$ is the pre-exponential factor, which takes into account the number of possible reactions per second (see for example Pawin *et al* [22]). $k_{\rm B}T = 0.034$ eV, where *k*_B_ is the Boltzmann constant and $T~=~400$ K is the reaction temperature suitable for aryl-aryl cross-coupling reactions that are catalysed by a metal surface or in solution [6, 13, 23–26]. In the type of self-assembly proposed, dehydrogenation is not required and therefore higher reaction temperatures have not been considered.

Although catalyst effects are not explicitly nor directly included, these can be added to first order by perturbing the activation energies [28, 29]. Hence, in this respect, $Ea_{AA}$ and $Ea_{BC}$ are chosen as free parameters in the range 0.01–1.0 eV corresponding to a possible molecular-reaction phase space for catalyst-assisted synthesis [6, 23, 26–28]. In terms of surface interactions, free diffusion across a homogeneous surface is also assumed so that the system is well mixed and coupling limited. This assumption is justified against studies that show the largest reaction barrier, and hence rate-limiting step, to be defunctionalization (e.g. dehalogenation, against aryl diffusion and aryl combination as per Björk *et al* 2013 [23]). Within this reaction space, conditions for producing high-quality nanographene with length-to-width tunabilty via directional growth will be determined. These reaction conditions will also be compared against published density functional theory studies to assess experimental feasibility and to propose the type of molecular functionalization (A)–(C) and catalyst.

Between 400 (coarse-grain limit) and 2000 (fine-grain limit) precursor molecules are used in each simulation run with the volume of the reaction chamber set so that an initial molecule concentration of 1000 molecules per litre is maintained. The rate of bond formation

6 \begin{align*} \displaystyle r = k[\,f][g] \label{rate1} \nonumber \end{align*}

is proportional to the molecule concentrations $[\,f]$ and $[g]$, with reactions between identical molecules containing a correction term to avoid double counting. For example, for two reacting *f* molecules,

7 \begin{align*} \displaystyle r = k\frac{[\,f][\,f - 1]}{2}. \label{rate2} \nonumber \end{align*}

Once formed, it is assumed that there is no mechanism for molecular aggregates to break apart.

At the start of this example simulation, functionalized tetrabenzanthracene and functionalized benzene are assigned to molecules *f* and *g*, respectively. The types of bonds between the molecules are first determined using the cross-correlation algorithm (section 2.1). In this case, *f*–*f* molecule binding is possible through eight different rotations and translations, each time forming two A–A bonds, whereas, for *f*–*g* (i.e. B–C) binding, bond formation is possible in eight different rotations. This information is contained in the *geometries of interaction* array, 
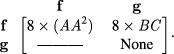


The calculated rate constants for these bindings are determined using the Arrhenius equation (equation (5)) and stored in the *rate constant* array $[k]$, 
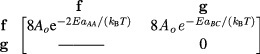
 with the molecule concentrations also stored. 
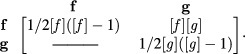


By obtaining the product of the elements in the above two arrays, a *binding rate* array can be determined for the rates of reaction,

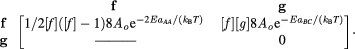


The sum of the upper quadrant elements of the *binding rate* array denotes the total reaction rate for the system until the next reaction is simulated. Within this time step, the reaction rates are normalized such that the probabilities for each reaction are in the range $(0, 1)$.

A probabilistic approach is chosen to stochastically select the next occurring reaction using the Gillespie algorithm [30]. Using this method, only interactions that are possible within the population at each reaction step are considered, thus forming a reaction network. Reactions are chosen stochastically using a pseudo random number generator to select from the probability-weighted list of reactions. Following these processes, the simulation timer is advanced by a characteristic time step of 120s representing the time during which an experimentalist could terminate the chemical synthesis process, thus completing the Gillespie simulation step.

The Gillespie approach decreases the computational search space so that fewer interactions are considered as the total number of reacting molecules decreases over time. In implementing the Gillespie algorithm, the relative likelihood of binding due to the mobility and size of the reacting components has not been considered. These constraints are in keeping with the reactions not being diffusion limited and in assuming a well-mixed homogeneous volume. Although a well-mixed system is typical for the Gillespie approach, the model can be extended to include diffusion [31, 32]. These extensions have not been explored here, but may be required for other types of reactions where the effect of surface directionality [33] and chemical reaction by-products [23] need to be considered. The simulation finishes when there are no possible reactions or when any further possible reactions are likely to take more than the characteristic time step of 120s. As the simulation is stochastic, 20 simulations have been run at each of the 100 sampling points in the $Ea = 0.01$–1.0 eV (coarse-grain) range to statistically represent the system behavior. For fine-grain sampling, a smaller activation energy space is explored with 40 simulations per sampling point.

**Figure 8.**
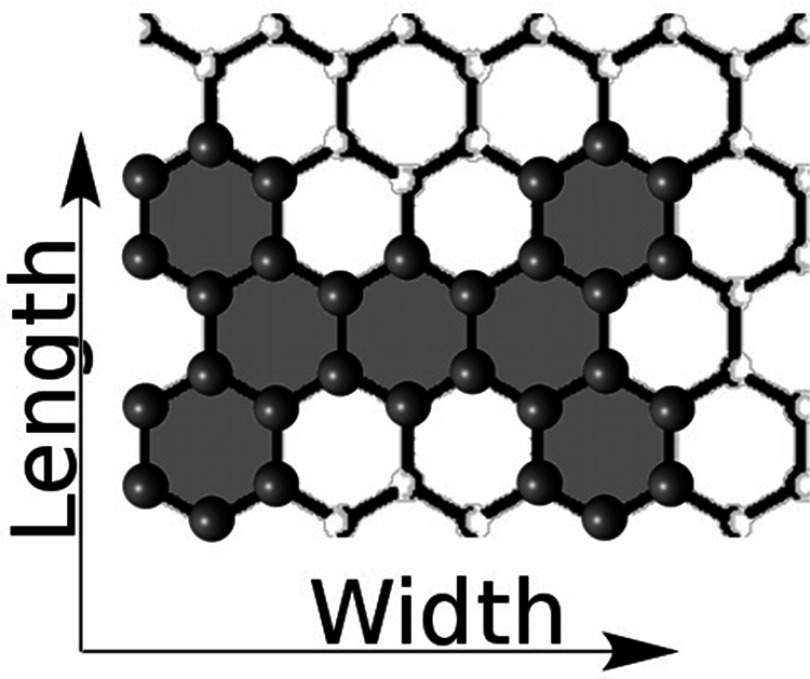
The width *W* and length *L* of the molecule precursors and products are defined by the maximum number of whole hexagons covered in each direction. In this case, $W=4$ and $L=3$. The number of whole benzene rings in the molecule ($N = 7$) is shaded.

At regular intervals during the simulation, the state of the system is saved so that the time-evolution and the reaction network can be analyzed. The synthesized molecular products are assessed using the parameters, length (*L*), width (*W*), and the number of whole benzene rings (*N*) in the molecule (figure 8). The length-to-width ratio

8 \begin{align*} \displaystyle R = \frac{L}{W}\label{ratio_eq} \nonumber \end{align*}

interrogates directional growth, and the area occupancy

9 \begin{align*} \displaystyle O = \frac{N}{N_I}\label{occupancy_eq} \nonumber \end{align*}

defines the *completeness* of the synthesis. Here, *N*_*I*_ is the number of whole benzene rings in an ideal synthesized system made from the available building blocks, which can fit into the $L \times W$ area. A *combination* score

10 \begin{align*} \displaystyle C = O \frac{L}{W} = OR \label{quality} \nonumber \end{align*}

is defined where $L &gt; 3$ and $W &gt; 4$, i.e. when there has been a binding event in both the A–A and B–C directions. Otherwise this parameter is set to zero when $L\leqslant 3$ and/or $W\leqslant 4$ and defines regions in the $Ea_{AA}$ versus $Ea_{BC}$ phase space where, on average, there is no growth, or uni-directional growth only, i.e. the latter being for $Ea_{AA} \gg Ea_{BC}$, and vice versa. Interrogation of the properties in equations (8)–(10) within the $Ea_{AA}$ versus $Ea_{BC}$ phase space will determine the optimal energetics to produce nanographene from non-trivial, directed self-assembly growth in both length and width dimensions, and with well-defined completion (area occupancy).

## 3. Results and discussion

**Figure 9.**
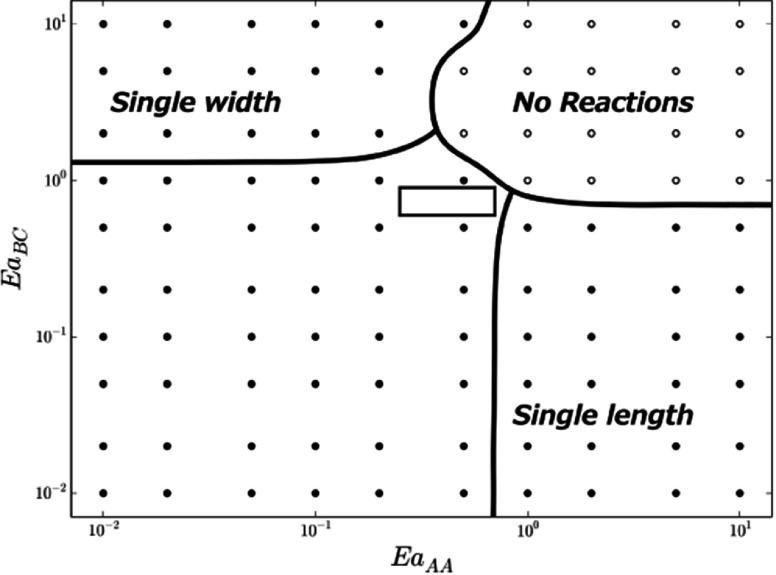
Schematic showing key regions for functionalized tetrabenzanthracene and benzene self-assembly at $T = 400$ K and $k_{\rm B}T=$ $0.034$ eV. $Ea_{AA}$ and $Ea_{BC}$ are the activation energies in eV for A–A and B–C bond formations (figure 1). Here, *single width* and *single length* refer to regions where nanographene products are predominantly of single tetrabenzanthacene unit width or length. The boxed region (center) is an area of interest for non-trivial two-dimensional growth that will later be explored in detail.

A schematic phase diagram corresponding to the activation bond energies $Ea_{AA}$ and $Ea_{BC}$ has been derived from the simulation results, with key regions defined in phase space for the expected type of reaction end products (figure 9). These regions can be explained in terms of the reaction energetics of the bond activation energies relative to $k_{B}T$ as per the Arrhenius equation (equation (5)). When both $Ea_{AA}$ and $Ea_{BC}$ are much greater than $k_{B}T$, then no reactions occur within the characteristic time step (120s) (top right, figure 9). When $Ea_{AA} \gg Ea_{BC}$, reaction products are produced that have single unit cell length, but variable width, and vice versa. Of interest is a region that occurs in the centre of figure 9 (boxed) where non-trivial two-dimensional growth occurs. This region will later be analyzed in detail.

**Figure 10.**
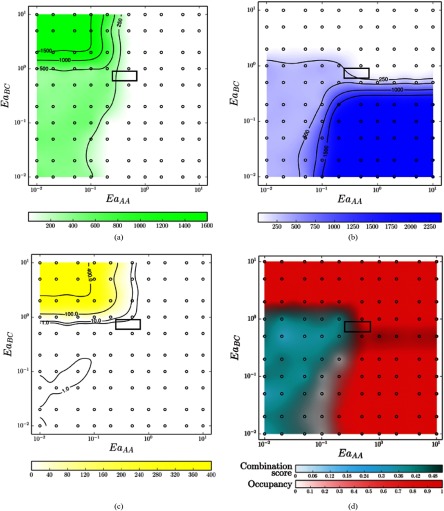
Coarse-grain phase diagrams for functionalized tetrabenzanthracene and benzene self-assembly at $T=400$ K. $Ea_{AA}$ and $Ea_{BC}$ are the activation energies in eV for A–A and B–C bond formations (figure 1). Simulations were conducted 20 times at each indicated sample point over the 0.01–1.0 eV ranges for $Ea_{AA}$ and $Ea_{BC}$ (100 sampling points in total). The phase diagrams for the mean values of the (a) length, (b) width, and (c) length-to-width ratio of the product nanographene systems are shown, as well as the (d) occupancy (i.e. *completeness*) and combination score. A non-trivial area relating to two-dimensional growth is indicated (central boxed area). (a) Mean length (*L*). (b) Mean width (*W*). (c) Mean length-to-width ratio (*R*). (d) Mean occupancy (*O*) and combination score (*C*).

Phase diagrams obtained via coarse-grain simulation show mean values for the length (figures 10(a) width (b)), length-to-width ratio (figure 10(c)), occupancy (i.e. degree of *completion*) and combination score (figure 10(d)) for the nanographene products. In the *No Reactions* region where $Ea_{AA}$ and $Ea_{BC}$ are $\gg$$k_{\rm B}T$ (see figure 9), there is evidence that the initiator molecules remain largely unreacted as the combination score $C=0$, i.e. in this case, $L \leqslant 3$ and $W \leqslant 4$, however, the area occupancy *O* remains maximal. Single-width and single-length regions are found where $Ea_{AA} \gg Ea_{BC}$ and $Ea_{AA} \ll Ea_{BC}$, respectively. These regions also have $C=0$ due to uni-directional growth (i.e. $L = 3$ or $W = 4$), but with high values of *O*, thus indicating good completion.

If both $Ea_{AA}$ and $Ea_{BC}$ are very low (bottom left of the phase diagrams in figure 10), then all possible reactions can occur very quickly resulting in largely uncontrolled and non-directional growth. At such low reaction energies, a single disordered nanographene product may be produced as the molecules non-discriminatorily bind to many points in the growing system. In general, regions that result in mostly non-directional growth are indicated where $W\sim L$, together with low occupancy *O* (blue dominant region in figure 10(d)). When both *C* and *O* are low (white region in figure 10(d)), there starts to be width-dominant growth $W&gt; L$, but with extremely poor completion *O* leading to a low combination score *C*. Ranges pertaining to the observables in equations (8)–(10) obtained using the coarse-grain sampling are summarized in table 1.

**Table 1.** Observable ranges for coarse-grain sampling as per figure 10.

Observable	Minimum value	Maximum value
Length (*L*)	3	1599
Width (*W*)	4	2399
Length-to-width ratio (*R*)	0.001	400
Area occupancy (*O*)	0.15	1.00

At the bottom of the length-dominated phase region (figure 10(a)), and at the top of the width-dominated phase region (figure 10(b)), there appears a band where the combination score *C* is high, extending from a region of length-dominant growth to width-dominant two-dimensional growth (see figure 10(d)). This region of transition is regarded as an area of interest for non-trivial directed self-assembly. Thus, to obtain nanographene products with good completeness and to avoid single unit cell dimension systems, the activation energies for $Ea_{AA}$ and $Ea_{BC}$ are set to 0.25–0.70 eV and 0.60–0.90 eV, respectively (boxed area in figure 10).

**Figure 11.**
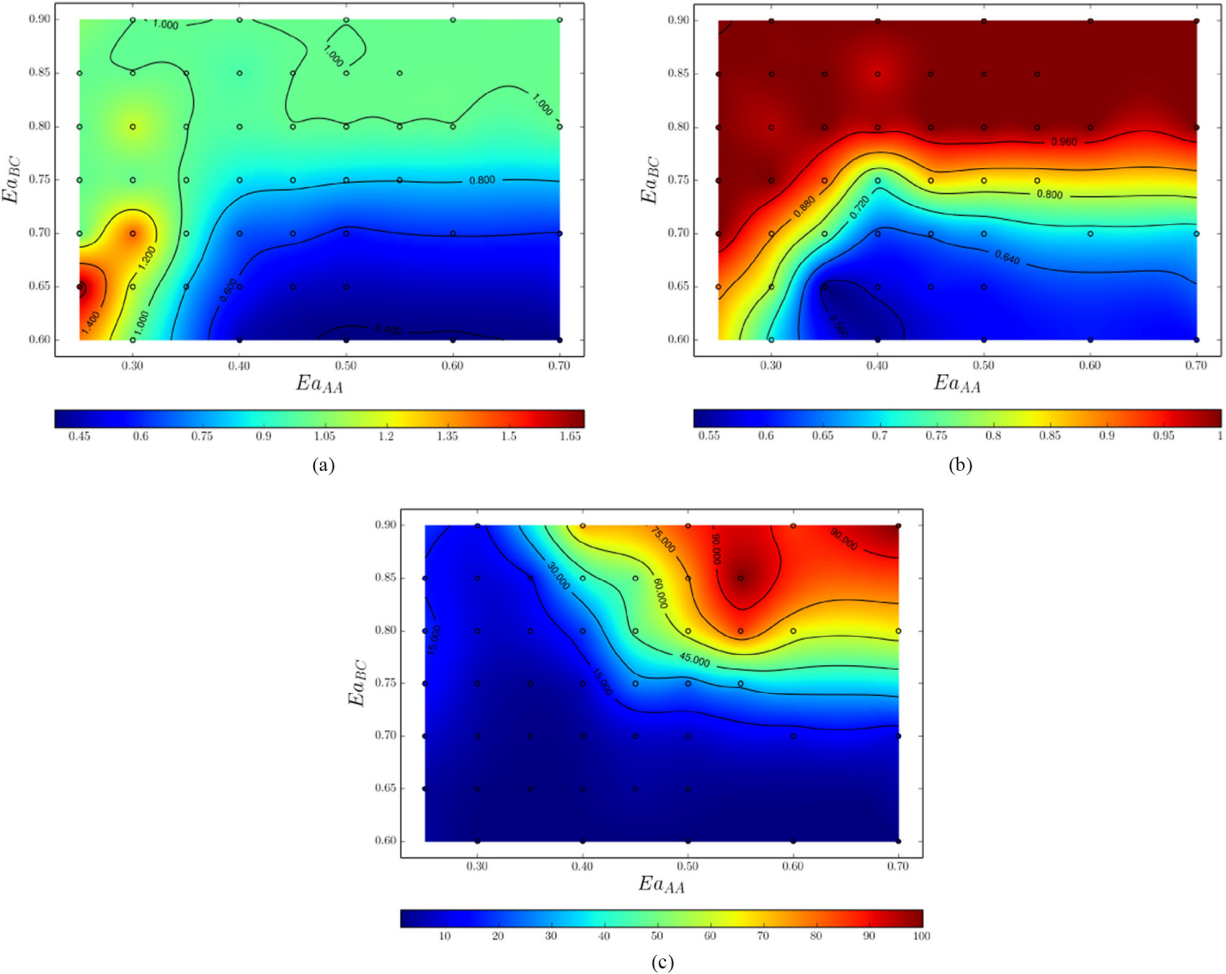
Phase diagrams for functionalized tetrabenzanthracene and benzene self-assembly using fine-sampling. The mean length-to-width ratio (a), area occupancy (b), and number of reaction products (c) are shown. $Ea_{AA}$ and $Ea_{BC}$ are the activation energies in eV for A–A and B–C bond formations. Simulations were conducted 40 times at each sample point in the $Ea_{AA}$ and $Ea_{BC}$ phase space. (a) Mean length-to-width ratio (*R*). (b) Mean area occupancy (*O*). (c) Mean number of reaction products (*N*_*P*_).

Fine-sampling from this reduced area (figure 11) indicates a much richer phase space than what was extrapolated to in the coarse-grain sampling simulations (see figure 10). Figure 11(a) shows the mean values for the length-to-width ratio *R* of nanographene products in the $Ea_{AA} = 0.25$–0.70 eV and $Ea_{BC} = 0.60$–0.90 eV ranges. Most of the nanographene products have relatively small defects as evidenced by high values of the area occupancy *O* over most of the reaction range (figure 11(b)). The small numbers of nanographene products (*N*_*P*_) in regions of high values of *O* indicates efficient synthesis (top left, figures 11(b) and (c)). Length-dominant growth ($R &gt; 1$) with molecular completion $O\geqslant0.72$ is seen when $Ea_{AA}$ is 0.25–0.30 eV and $Ea_{BC}$ is 0.60–0.70 eV. Width-dominant growth ($R &lt; 1$) occurs from $Ea_{AA}\simeq~0.35$–0.70 eV and $Ea_{BC}\simeq~0.60$–0.75 eV, but with reduced molecular completion (*O* ranging from 0.56 to 0.80).

**Figure 12.**
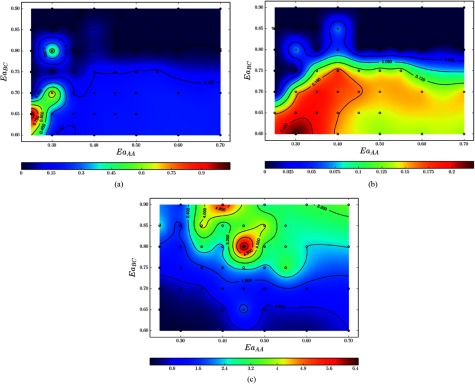
Phase diagrams for the standard error associated with the mean observable values for the (a) length-to-width ratio, (b) area occupancy and (c) number of reaction products for the tetrabenzanthracene and benzene self-assembly as per figure 11. Simulations were conducted 40 times at each sampling point indicated in the $Ea_{AA}$ and $Ea_{BC}$ phase space. (a) Standard error for the mean length-to-width ratio. (b) Standard error for the mean area occupancy. (c) Standard error for the mean number of reaction products.

A statistical representation of these simulations provides further insight and interpretation of the results as indicated by the standard error of the mean taken over the 40 measurements per sampling point (figure 12). For example, in the region of length-dominant growth (bottom left, figure 11), there occurs a reduced area occupancy score *O* and number of products *N*_*P*_ indicating that molecular bonds are produced quickly. As there are fewer constraints on unfavorable bond formation, evidence of uncontrolled synthesis can also be found in the correspondingly high standard error in *R* and *O* (figures 12(a) and (b)). In the width-dominant region ($Ea_{AA}\simeq~0.35$–0.70 eV and $Ea_{BC}\simeq~0.60$–0.75 eV), the standard error associated with these observables is comparatively lower.

Although candidate regions in the $Ea_{AA}$ and $Ea_{BC}$ phase-space show length-dominant and width-dominant, i.e. *directional* growth, the results indicate that further optimization is needed to achieve length-to-width tunability and to reduce defects, thus improving the quality, efficiency and predictability of the synthesis process. Modifying the bond activation energies by catalyst intervention may be one mechanism to achieve this aim. Another approach would be to change how the reaction is started such that the initial mixing of the initiator molecules is a possible experimental variable. Thus, the simulation could be modified by introducing different reactants at varying times to influence the production of low-defect nanographene with length and/or width tunabilty.

To perform this test, a point in the $Ea_{AA}$ versus $Ea_{BC}$ phase space is selected where the variability of the length-to-width ratio *R* is investigated as a function of the rate of functionalized benzene addition to the reaction cell. The activation energies $Ea_{AA}= 0.30$ eV and $Ea_{BC}= 0.75$ eV are chosen as the initial results showed good molecular completion (high *O*) and efficient synthesis with low *N*_*P*_ and low corresponding standard error for all quantities measured (figures 11 and 12). Although the mean length-to-width ratio *R* of the products was  ∼1.0, the result at $Ea_{AA}= 0.30$ eV and $Ea_{BC}= 0.75$ eV is in a region close to the length-dominant and width-dominant regimes. Thus, perturbing the reaction conditions may influence the system towards directional growth with good completion (i.e. to produce low defect products).

**Figure 13.**
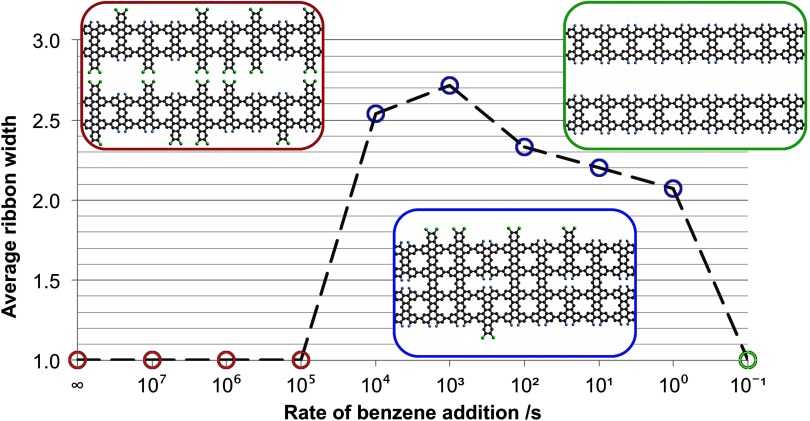
Functionalized benzene is introduced into the reaction chamber at different rates, or all at once ($\infty$), at $Ea_{AA} = 0.30$ eV and $Ea_{BC} = 0.75$ eV. Average nanographene widths relative to the width of a single tetrabenzanthracene unit are shown to be dependent on these rates. Quicker rates (red) yield predominantly single-width systems. For slow rates (green), the reaction is terminated before any benzene is bound to the ribbon. At intermediate rates (blue), wider nanographene systems form. The inset images show representative systems that are produced.

Variable mixing rates for the addition of functionalized benzene at the start of each simulation were tested, with the addition of benzene continuing until its amount became equal to tetrabenzanthracene. The characteristic time step of 120 s was maintained. The results show that the width of the nanographene products can be tuned as a function of the simulated functionalized benzene addition rate (figure 13). If all of the functionalized benzene is added at the start of the reaction, or very quickly, then only single-width nanographene systems are formed on average (i.e. growth in the length-direction is preferred) (figure 13 (red)). When the addition-rate approaches 10^3^ ${\rm s}^{-1}$, wider nanographene products are produced (figure 13 (blue)). At low addition rates (around $10^{-1}$ ${\rm s}^{-1}$) there is not enough functionalized benzene to start a reaction before the simulation terminates, thus producing single-width nanographene only (figure 13 (green)).

At faster addition rates, single-width growth of nanographene occurs due to larger amounts of benzene being available to cap the tetrabenanthracene. This effect makes it statistically unfavorable for molecular reactions as benzene-benzene structural clashes impede further width growth (figure 13, red-box inset). At lower addition rates, slower benzene addition can facilitate width growth (figure 13, blue-box inset). At the chosen bond activation energies, the binding of benzene to tetrabenzanthracene is not intrinsically unfavourable. Rather, the lower effective concentration of benzene corresponds to a lower rate of bond formation (equation (6)), enabling wider nanographene systems to form. Notably, these wider nanographene products also have low numbers of defects with the area occupancy *O* being maintained above 90% (figure 13, blue-box inset). Closing all of the gaps in the nanographene systems could be facilitated as a further simulation step after the model system has equilibrated through the addition of other functionalized molecules.

The results in figure 13 predict that the nucleation of self-assembly, in this and similar systems, could be best performed with the gradual introduction of initiator species into the reaction chamber (i.e. by using a time-dependent experimental protocol). Further simulations are needed to determine whether this is essential to achieve optimal self-assembly under different reaction conditions, hence, parameterization of the model. The required time to halt assembly (included in the model as the characteristic time step of 120 s) is proportional to the activation energies, and this could also be altered to check for more efficient simulation conditions.

**Figure 14.**
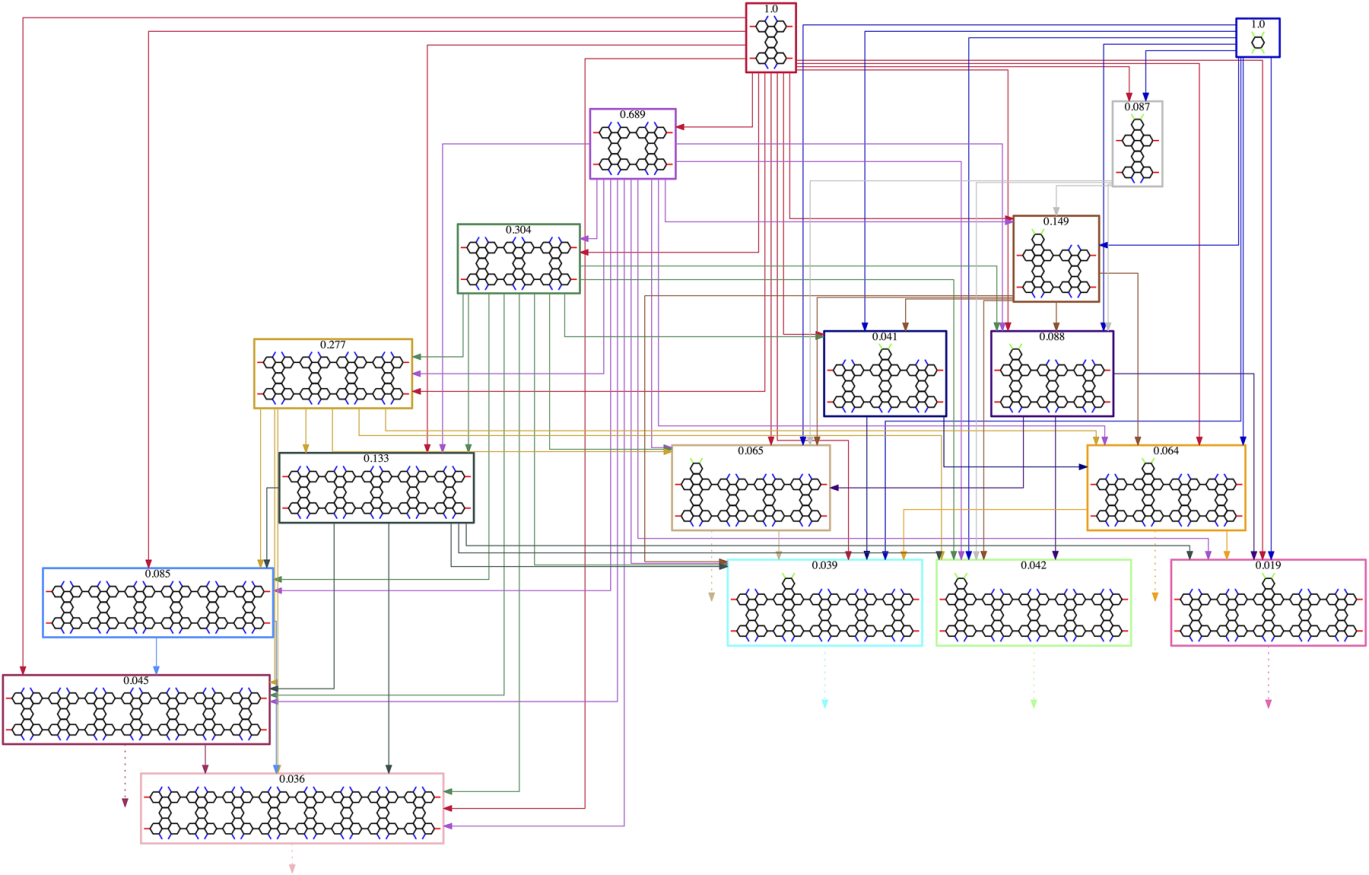
Example of the reaction network and intermediate molecules obtained in the simulation at $Ea_{AA} = 0.30$ eV and $Ea_{BC} = 0.75$ eV. The flow parameter *f* is shown for each molecule in the network and provides a time-averaged measure of the proportion of the initiator molecules aggregating to specific intermediate molecules. Full access to the network of reaction pathways and the ability to interrogate intermediates allows for future design capability. In this respect, the chemical reaction can be engineered and then directed to favor chosen synthesis pathways and to produce a high yield of desired reaction products.

A benefit of the kinetic self-assembly method is the ability to interrogate the intermediates and reaction pathways from each simulation. Figure 14 provides an example of a reaction network for the simulation of functionalized benzene and tetrabenzanthracene synthesis at $Ea_{AA}=0.30$ eV and $Ea_{BC}=0.75$ eV. To quantify the intermediate synthesis pathways in the simulation, a flow parameter *f* is computed, which is defined as the occurrence of a specific intermediate molecule multiplied by the number of initiator molecules that have been used to produce it. Thus, the flow parameter provides a time-averaged measure of the proportion of initiator molecules that aggregate into specific intermediate molecules normalized to the number of initiators at the start of the synthesis. For example, both functionalized tetrabenzanthracene and benzene have values of $f=1.0$ at $t=0$ (figure 14).

Interrogation of the reaction network and study of the flow metric as a function of the synthesis conditions could lead to the engineering of certain synthesis pathways and experiments that target specific (i.e. *directed*) molecular growth or reaction products with desired properties. Coupling the bottom-up kinetic self-assembly model dynamically with rapid simulation to determine the properties of the products, such as the generalized tight-binding method [18] (figure 2), could enable further real-time selectivity of the chemical processes and outputs.

We have focused this paper on demonstrating a novel computational method for directed nanographene self-assembly, which is physically transparent with the example results from the model providing an indication of its potential use in predictive simulation. A region in the activation energy range of interest $Ea_{AA} = 0.25$–0.70 eV and $Ea_{BC} = 0.60$–0.90 eV was found for directed self-assembly of functionalized tetrabenzanthracene and benzene resulting in length-to-width ratio tunability and controlled quality of the reaction products. The defunctionalization step (assumed to be the rate limiting step), which occurs twice for the A–A reaction and four times for the B–C reactions leads to energy barriers of $Ea_{AA}/2$ and $Ea_{BC}/4$ assumed for each point defunctionalization. This results in reaction energetics ranging from  ∼0.13–0.35 eV for each A-atom defunctionalized and  ∼0.15–0.23 eV for each B- or C-atom defunctionalized (assumed to be energetically equal).

Current density functional theory literature shows that the activation energy barrier for dehalogenation is surface catalyst specific, for example, the Ullmann mechanism for dehalogenation of aryl halides can occur with an activation energy barrier of  ∼0.4 eV for a copper surface [6, 23]. Lower activation energy barriers are possible for other surfaces, such as palladium [34], or if other conditions for site activation are considered, such as the oxidative state, or metal promotion of the surface [35]. Comparison of the reaction energetics determined from this work against the available density functional theory data (referenced here) suggests the proposed synthesis may be experimentally plausible via halogen functionalized aryl-aryl cross-coupling and noble metal surface-assisted catalysis. A more precise prediction would require density functional calculations of the energy barriers (both coupling and diffusion) in relation to the specific system studied with various functionalization and catalytic surfaces tested. In this respect, the proposed kinetic self-assembly method could be used to determine new synthesis processes and for materials discovery of novel nanographene.

## 4. Conclusion

An algorithm for simulating nanographene production using a predictive, kinetic self-assembly method has been proposed. The kinetics-based model allows for refinement of existing protocols (initiator molecules, bond energies, reaction temperature), and the ability to introduce new experimental protocols (surface interactions, etc). Nanographene systems with desired characteristics via the studied metrics (length-to-width ratio, degree of completeness, and number of reaction products), and required properties, can be searched for by simulating different synthesis conditions. Through these metrics and their standard errors, the reproducibility of the experiments can also be assessed.

As an exemplar test-case, the self-assembly of functionalized benzene and tetrabenzanthracene was used to demonstrate the functioning of the model. A region in the bond activation energy phase space was determined, which has potential for *directed* self-assembly growth via tuning of the length-to-width ratio of the product molecules. Further control of the length-to-width ratio was shown by the slow addition of functionalized benzene into the reaction chamber. We provided an assessment of the reaction conditions against available published results on synthesis energetics, which suggest that the synthesis may occur via halogen-functionalized aryl-aryl cross-coupling and nobel metal assisted catalysis. Detailed studies via density functional theory on the proposed system would be needed to confirm these predictions. A benefit of the proposed application of kinetic self-assembly is that full access to the reaction network provides the possibility to tune the synthesis conditions to bias specific reaction pathways and reaction products. Dynamic coupling of the self-assembly method to a density functional theory-informed tight-binding approach could facilitate rapid simulation-driven nanographene design with prediction of device properties. Future work will include more specific conditions, such as surface interactions, therefore providing further realistic refinements. Although only shown in a minimal model capacity, the simulations suggest an efficient and physically transparent approach towards bottom-up engineering of nanographene.
